# Envelope Protein Mutations L107F and E138K Are Important for Neurovirulence Attenuation for Japanese Encephalitis Virus SA14-14-2 Strain

**DOI:** 10.3390/v9010020

**Published:** 2017-01-21

**Authors:** Jian Yang, Huiqiang Yang, Zhushi Li, Wei Wang, Hua Lin, Lina Liu, Qianzhi Ni, Xinyu Liu, Xianwu Zeng, Yonglin Wu, Yuhua Li

**Affiliations:** 1 Department of Viral Vaccine, Chengdu Institute of Biological Products Co., Ltd., China National Biotech Group, Chengdu 610023, China; jiany74@163.com (J.Y.); yang-anan@163.com (H.Y.); changdc123@sina.com (Z.L.); suntina926@163.com (W.W.); scciqlh@126.com (H.L.); linaliu@163.com (L.L.); nqz1986@126.com (Q.N.); zengxw64@163.com (X.Z.); 2 Department of Microbiology and Immunology, North Sichuan Medical College, Nanchong 637007, China; 3 Department of Arbovirus Vaccine, National Institutes for Food and Drug Control, Beijing 100050, China; xinyuliu@hotmail.com; 4 China National Biotech Group, Beijing 100029, China; wuyonglin@sinopharm.com; 5 State Key Laboratory of Biotherapy and Cancer Center, West China Hospital, Sichuan University and Collaborative Innovation Center for Biotherapy, Chengdu 610000, China

**Keywords:** attenuation mechanism, Japanese encephalitis virus, SA14-14-2, neuroinvasiveness, neurovirulence

## Abstract

The attenuated Japanese encephalitis virus (JEV) strain SA14-14-2 has been successfully utilized to prevent JEV infection; however, the attenuation determinants have not been fully elucidated. The envelope (E) protein of the attenuated JEV SA14-14-2 strain differs from that of the virulent parental SA14 strain at eight amino acid positions (E107, E138, E176, E177, E264, E279, E315, and E439). Here, we investigated the SA14-14-2-attenuation determinants by mutating E107, E138, E176, E177, and E279 in SA14-14-2 to their status in the parental virulent strain and tested the replication capacity, neurovirulence, neuroinvasiveness, and mortality associated with the mutated viruses in mice, as compared with those of JEV SA14-14-2 and SA14. Our findings indicated that revertant mutations at the E138 or E107 position significantly increased SA14-14-2 virulence, whereas other revertant mutations exhibited significant increases in neurovirulence only when combined with E138, E107, and other mutations. Revertant mutations at all eight positions in the E protein resulted in the highest degree of SA14-14-2 virulence, although this was still lower than that observed in SA14. These results demonstrated the critical role of the viral E protein in controlling JEV virulence and identified the amino acids at the E107 and E138 positions as the key determinants of SA14-14-2 neurovirulence.

## 1. Introduction

The Japanese encephalitis virus (JEV) belongs to the *Flavivirus* genus and causes frequent endemic and epidemic infections in Asia, with JEV infection leading to acute encephalitis in humans and resulting in high mortality rates. The wild-type JEV SA14 strain was isolated from mosquitoes in Xi’An, China in 1954, and the attenuated JEV SA14-14-2 strain was obtained by serial passages of the JEV SA14 strain in mouse brain and primary hamster kidney (PHK) cells, followed by purification by plaque screening [1]. The purified SA14-14-2 strain was used to produce the attenuated live Japanese encephalitis (JE) vaccine for humans, with >600 million doses of this vaccine being administered in China and other countries in Southeast Asia, including Korea, Nepal, India, and Thailand, since 1989. The safety and efficacy of this vaccine have been well demonstrated by clinical data [2], and on 10 September 2013, it passed World Health Organization prequalification and was entered into the list of vaccines available for international purchase. As with all attenuated live viral vaccines, its reversion to virulent status remains a concern. This study explored the molecular mechanisms underpinning the attenuated neurovirulence of the live JE vaccine (SA14-14-2) by reverting specific amino acids in the SA14-14-2 envelope (E) protein to their counterparts in the parental virulent strain (SA14) and testing the virulence of the revertant viruses.

Our findings indicated no neurovirulence observed in adult mice inoculated intracerebrally (i.c.) with attenuated JEV SA14-14-2 at 10^6^ plaque-forming units (PFUs), as compared with mice inoculated with the parental strain, which caused 100% mortality in mice within 1 week. The marked virulence attenuation of JEV SA14-14-2 is believed to result from specific substitutions at 24 amino acid positions, including eight amino acid mutations in the E protein, throughout the viral genome [3,4,5], as well as mutations in nonstructural proteins [6]. However, the specific mutations that determine the attenuated SA14-14-2 phenotype remain unknown.

The attenuated yellow fever virus (YFV) 17D strain differs from its parental Asibi strain by 32 amino acid substitutions. Among these, 12 mutations are located in the E protein. Remarkably, as few as one mutation (E303) in the E protein can change the attenuated phenotype of the Asibi strain [7]. The crucial role of amino acid mutations in the E protein, associated with attenuation, was reported in other attenuated viral vaccines, including the chimeric yellow fever-dengue 1 vaccine virus [8]. That study hypothesized that the attenuated phenotype of the JEV SA14-14-2 strain might also be attributed to specific mutations in the E protein. Here, we investigated the roles of five amino acid residues (E107, E138, E176/177, and E279) in the E protein in the attenuated strains, as compared with the virulent parental strain (Table 1), followed by an assessment of the neurovirulence and neuroinvasiveness of these revertants in mice. Our results demonstrated that amino acids at the E138 and E107 positions played key roles in neurovirulence attenuation in the JEV SA14-14-2 strain.

## 2. Materials and Methods

### 2.1. Cells, Plasmids, and Viruses

BHK-21 cells (CCL-10; American Type Culture Collection, Manassas, VA, USA) were cultured in an Eagle minimum essential medium (MEM; Gibco; Thermo Fisher Scientific, Waltham, MA, USA) supplemented with 10% heat-inactivated fetal bovine serum. The multiple-cloning site of the low-copy plasmid pACNR was modified to contain the restriction sites *Asc*I, *Kas*I, *Bgl*II, *BspE*I, *BamH*I, *Bcl*I, *Xba*I, and *Xho*I. The JEV SA14-14-2 strain was generated in PHK cells isolated from 9- to 10-day-old specific pathogen-free (SPF) hamsters at the Chengdu Institute of Biological Products Co., Ltd. (Chengdu, China).

### 2.2. DNA Cloning

The RNA of the JEV SA14-14-2 strain was extracted using a High Pure viral RNA kit (Roche, Basel, Switzerland), and cDNA was synthesized by reverse transcription (RT) using SuperScript III reverse transcriptase (Invitrogen, Carlsbad, CA, USA). Briefly, 20 ng RNA was mixed with 10 pmol 3′-terminal primers, heated for 5 min at 65 °C, cooled on ice for 1 min, and then incubated with SuperScript III in the recommended buffer for 1 h at 55 °C, followed by heating to 70 °C for 15 min. cDNA amplification was performed with the phusion polymerase (New England Biolabs, Ipswich, MA, USA) using a touchdown polymerase chain reaction (PCR) program: one cycle at 98 °C for 1 min, 10 cycles at 98 °C for 15 s, 58.5 °C to 53.5 °C for 15 s, and 72 °C for 3 min, followed by 20 cycles at an annealing temperature of 53.5 °C and elongation for 10 min at 72 °C. PCR products were purified using a DNA purification kit (Qiagen, Hilden, Germany) and cloned into the pGEM-T easy vector (Promega, Durham, NC, USA). The correct clones were identified by DNA sequencing.

All plasmids were constructed using two-plasmid systems as described previously [9,10]. One plasmid contained the 5′ terminal 3.4-kb cDNA and the other contained the 3′ terminal 7.6-kb fragment of the SA14-14-2 strain. The first fragment (1–476 nt) contained *Asc*I and *Kas*I restriction sites [11] and was cloned into the low-copy plasmid pACNR. The second fragment, from position 476 to 2654, and the third fragment, from position 2640 to 3446, were inserted into the *Kas*I/*Bgl*II and *Bgl*II/*BspE*I sites, respectively, to generate the plasmid pACNR-5′JEV (harboring the 5′ terminal 3.4-kb fragment). The fourth fragment, from position 3444 to 5581; the fifth fragment, from position 5575 to 7092; the sixth fragment, from position 7086 to 9136; and the seventh fragment, from position 9130 to 10977, were cloned into the pACNR to create the plasmid pACNR-3′JEV (containing the 3′ terminal 7.6-kb fragment). This 7.6-kb fragment of JEV was then inserted into the plasmid pACNR-5′JEV to create the plasmid pACNR-JEV containing the full-length cDNA of JEV SA14-14-2. Mutations in the E protein gene were generated by PCR-based site-directed mutagenesis, and all plasmids were sequenced to verify the engineered mutations.

### 2.3. In Vitro RNA Transcription, Transfection, and Viral Recovery

The pACNR-JEV plasmid was linearized by restriction digest using *Xho*I and used as a template for in vitro transcription. The RNA used for transfection was synthesized using the RiboMAX large-scale RNA production system Sp6 kit (Promega) in the presence of Ribo m7G cap analog (Promega). Reaction products were treated with DNase I (RQ1 RNase-free DNase; Promega), followed by purification with the RNeasy mini kit (Qiagen). BHK-21 cells were washed twice with cold phosphate-buffered saline, then 4 × 10^6^ cells in 200 µL were mixed with the synthesized RNA in vitro (1 µg) and pulsed at 140 V for 25 ms using a Gene Pulser II apparatus (Bio-Rad, Hercules, CA, USA). Transfected BHK-21 cells were cultivated at 37 °C in a 5% CO_2_ incubator, and the viruses were harvested at day 5 post-transfection upon observation of the cytopathic effect. The harvested viruses were passaged two additional times in BHK-21 cells, titrated for the plaque assay, and stored at −80 °C until further use.

### 2.4. Nucleotide Sequencing of the Revertant Viruses

Briefly, viral RNA was extracted from the recovered viruses using the High Pure viral RNA kit (Roche). cDNA from position 468 to 2667 containing the prM/E protein gene was synthesized by RT, followed by the amplification of the prM/E fragment using the phusion polymerase (New England Biolabs). The PCR products were purified using the QIAquick gel extraction kit (Qiagen) and sequenced to determine the consensus sequence (Invitrogen).

### 2.5. Growth Analysis of Revertants and Control Viruses

BHK-21 cells were infected with the revertants or control viruses at a multiplicity of infection of 0.5. After 1 h of absorption at 37 °C, viral inocula were removed, and 20 mL MEM containing 2% inactivated newborn calf serum was added. Culture supernatant (1 mL) was collected every 24-h post-infection for 96 h. Titers of the collected viruses were determined as described for the plaque assay.

### 2.6. Mouse Experiments

To assess and compare neurovirulence, groups (*n* = 4) of 3-week-old SPF Kunming mice were inoculated with 0.03 mL of 10-fold dilutions of the revertants or the control viruses by the i.c. route, and inoculated mice were monitored for 14 days. All moribund mice were euthanized, and the median lethal dose (LD_50_) was determined by the Reed and Muench calculation. Neurovirulence results for each virus were recorded as LD_50_ (log_10_PFU; the viral dose capable of inducing 50% mortality). Neuroinvasiveness was measured by inoculating 3-week-old SPF Kunming mice with 0.1 mL of 10-fold dilutions of the revertants or the control viruses by the subcutaneous (s.c.) route, and the neuroinvasive results were also recorded as LD_50_ (log_10_PFU). The average survival time (AST) was determined by inoculating 0.03 mL of viruses containing equal plaque titers (5.18 log_10_PFU) in another group of mice (*n* = 6) by the i.c. route. Mice in a moribund condition were euthanized and scored as deaths.

### 2.7. Statistical Analysis

Statistical analysis of the AST was performed using analysis of variance, and a *p* < 0.05 was considered statistically significant. All analyses were performed using SPSS software version 17.0 (SPSS, Inc., Chicago, IL, USA).

### 2.8. Ethical Approval

The experimental protocols involving mice were approved by the Experimental Animal Welfare and Ethical Committee of the National Institutes for Food and Drug Control, China.

## 3. Results

### 3.1. Construction of Infectious JEV Full-Length cDNA Clones Containing Specific Reverse Mutations in the E Protein

All pACNR-JEV plasmids containing specific mutations were verified by sequencing, and the viruses used for testing were amplified by three passages in the BHK-21 cells. The E protein-coding region of each virus was sequenced an additional time, with the results confirming that the sequences of all engineered plasmids and revertant viruses were correct and that no new mutations had been introduced.

### 3.2. Growth Analysis of Revertants and Control Viruses

**Figure 1:**
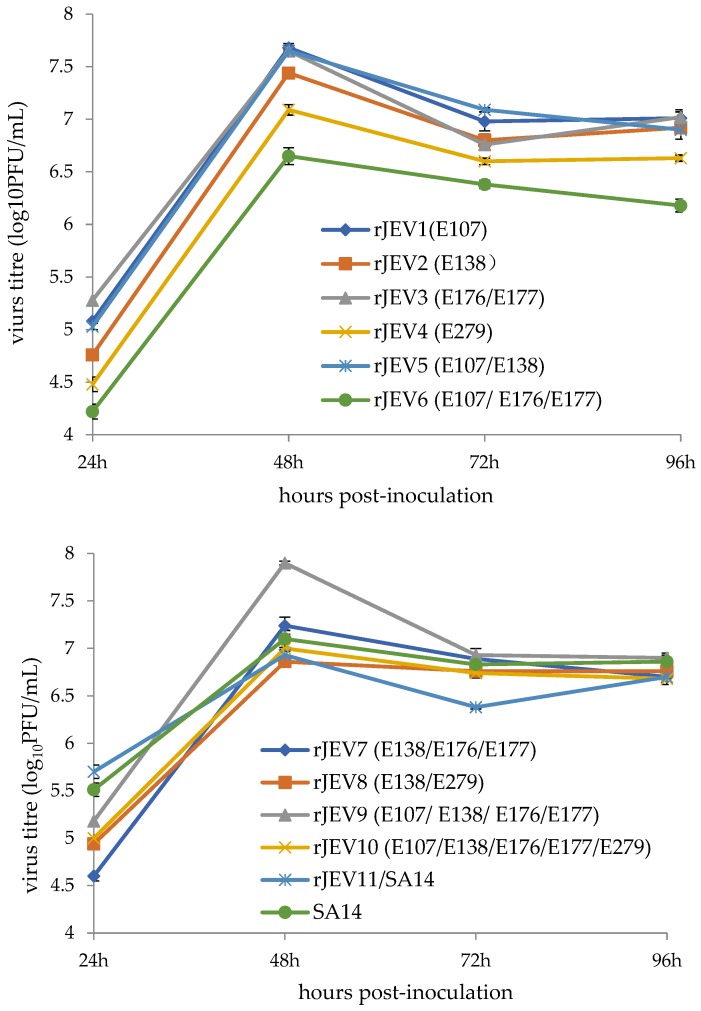
Growth curves of the revertants and the control viruses.

One mechanism of viral attenuation involves crippled viral replication [12]; therefore, the effects of reverse mutations on JEV replication were measured by infecting BHK-21 cells, followed by a determination of the production of revertants and control viruses at different time intervals following infection. Growth-curve results showed that all viruses exhibited similar replication capacities, although the SA14 virus replicated at a modestly faster rate, with 5.7 log_10_PFU/mL at 24-h post-infection, which was higher than the other viruses tested. However, the peak SA14 titer was not the highest among all viruses, which was likely due to the highest SA14 titer not being collected at the denoted time points (Figure 1).

**Figure 2:**
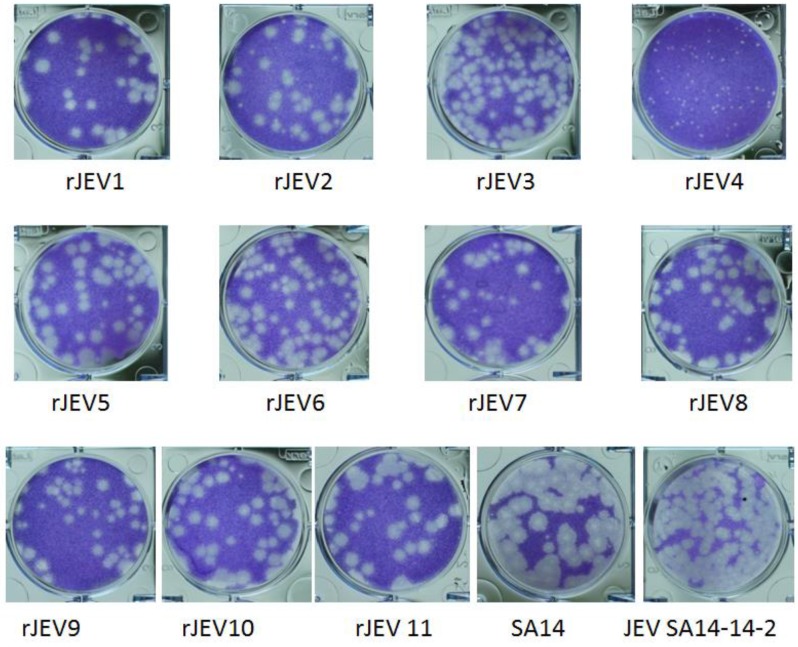
Plaque sizes of the revertants and the control viruses.

Additionally, analysis of the plaque sizes of all viruses revealed that those of SA14 (2–3 mm) were larger than those of SA14-14-2 (1–2 mm) and the other viruses (1–2 mm). We observed no significant difference in plaque size between SA14-14-2 and all the revertant viruses, except for that of rJEV4 (E279) (0.5–1 mm), which was significantly smaller than those of the other viruses (Figure 2).

### 3.3. Mutation at Residue E138 in Combination with E107 Is Critical to the Attenuated Neurovirulence of JEV SA14-14-2

To determine the amino acids in the E protein that attenuate JEV SA14-14-2 neurovirulence, we measured the LD_50_ (log_10_PFU) values of all the revertant viruses (Table 2), with low LD_50_ (log_10_PFU) values indicating high degrees of neurovirulence. Among revertants containing a single amino acid substitution, rJEV1 (E107) and rJEV2 (E138) exhibited lower LD_50_ (log_10_PFU) values as compared with that of the SA14-14-2 virus, whereas the revertant viruses rJEV3 (E176/E177) and rJEV4 (E279) exhibited similar LD_50_ (log_10_PFU) values to that of SA14-14-2. Among these four revertants, rJEV2 (E138) exhibited the lowest LD_50_ (log_10_PFU) value, indicating the highest degree of neurovirulence, followed by rJEV1 (E107). The reverse mutation of E138 in combination with E107 significantly decreased the LD_50_ (log_10_PFU) value as compared with those of rJEV1 (E107) and rJEV2 (E138), and the LD_50_ (log_10_PFU) value of rJEV10 (E107, E138, E176/177, and E279; 1.43) was slightly lower than that of rJEV9 (E107, E138, and E176/177; 1.99). The rJEV11/SA14 virus, wherein the E protein of SA14-14-2 was replaced with the E protein of wild-type SA14, exhibited the lowest LD_50_ (log_10_PFU) value (0.66) among all the revertants, although it was still higher than that of the virulent wild-type SA14 virus (−0.92). These findings suggested, that among the five tested amino acid residues, E138 and E107 played the most important roles in the attenuation of SA14-14-2 neurovirulence.

### 3.4. Reverse Mutations in the E Protein Increased the Mortality and Decreased the AST of I.C.-Inoculated Mice

The virulence phenotype of the revertant viruses was further evaluated by determining the mortality and AST of mice inoculated by the i.c. route with 5.18 log_10_PFU revertant virus (Table 3). Mice inoculated with rJEV2, rJEV5, rJEV7, rJEV8, rJEV9, rJEV10, rJEV11, or SA14 exhibited 100% mortality, whereas rJEV1 or rJEV3 inoculation resulted in 83.3% and 16.7% mortality, respectively, and SA14-14-2 or rJEV4 (E279) inoculation resulted in 0% mortality. These results showed that the E138 and E107 residues were more important than E279 and E176/177 at effecting SA14-14-2 virulence. The wild-type SA14 group exhibited the shortest AST (4 days), followed by the rJEV11 group (E107/E138/E176/177/E264/E279/E315/E439), with an AST of 5 days (*p* ≤ 0.05, compared with SA14). The AST of mice inoculated with rJEV2 (E138) exhibited an AST of 6 days, and the ASTs of the rJEV1 (E107) and the rJEV6 (E107/E176/177) groups were 6.6 and 9 days, respectively (*p* ≤ 0.05, comparing rJEV1 to rJEV6). The rJEV3 group (E176/177) exhibited the longest AST of 11 days. These results demonstrated that the E138 residue was a greater determinant of attenuated SA14-14-2 virulence, as compared with other residues in the E protein.

### 3.5. Effects of Specific Reverse Mutations on JEV SA14-14-2 Neuroinvasiveness

The neuroinvasiveness of all the revertants was tested using the same protocol as that used to test neurovirulence, except that the mice were inoculated by the s.c. route (Table 4). The LD_50_ (log_10_PFU) values of all the revertants containing single amino acid substitutions were similar to that of the attenuated SA14-14-2 strain, whereas the other revertants showed lower LD_50_ (log_10_PFU) values than that of SA14-14-2. The LD_50_ (log_10_PFU) values of the mice inoculated with rJEV5 (E107 and E138), rJEV7 (E138 and E176/177), or rJEV8 (E138 and E279) were ≥6.54, 5.76, and 6.01, respectively, suggesting that the E107 revertant mutation combined with E138 did not show the same synergistic effect as observed in the neurovirulence test. The LD_50_ (log_10_PFU) value of mice inoculated with rJEV10 (E107/E138/E176/E177/E279) was slightly higher than that of rJEV9 (E107/E138/E176/E177)-inoculated mice, and rJEV9 (E107/E138/E176/E177)-, rJEV10 (E107/E138/E176/E177/E279)-, and rJEV11/SA14-inoculated mice exhibited low LD_50_ (log_10_PFU) values of 5.40, 5.53, and 3.17, respectively. Furthermore, the LD_50_ (Log_10_PFU) value of mice inoculated with rJEV11 (rJEV11/SA14) was higher than that of mice infected with virulent SA14, indicating that other regions in the JEV genome also contributed to the neuroinvasive phenotype.

## 4. Discussion

Reverse genetics is a powerful tool for studying the replication, virulence attenuation, and gene functions of positive-strand RNA viruses. The key step in this strategy involves constructing stable cDNA clones containing the full-length viral sequence. However, constructing the full-length cDNA clone of the JEV SA14-14-2 strain was hindered by the instability and toxicity of some gene products in *Escherichia coli* [13,14,15]. Previous studies utilized two strategies to overcome these hurdles. One was an in vitro ligation approach [16] and the second involved using low-copy plasmids, such as those containing artificial bacterial chromosomes, to stabilize the full-length cDNA of JEV [17]. Here, a different low-copy plasmid (pACNR) was employed to stably maintain the full-length cDNA of the infectious JEV. To generate a marker in the recombinant viruses, a silent mutation was inserted at nucleotide 473 (from A to C) that also created a new restriction site (*Kas*I) for DNA cloning. This genetic marker allowed confirmation that the recovered viruses were derived from the infectious cDNA. Furthermore, this cDNA cloning system previously enabled the mechanistic study of the virulence attenuation of *Flaviviruses* and the development of other *Flavivirus* vaccines [18].

Several studies reported nucleotide changes potentially underlying the attenuated phenotype of the JEV SA14-14-2 strain through comparisons with its parental strain SA14 [3,4,19]. Major nucleotide changes in *Flavivirus* E proteins responsible for viral neurovirulence were also revealed by comparing the JEV AT31 strain with its attenuated derivative [20] and between YFV (the Asibi train) and its attenuated 17D strain [21]. The results of mouse-infection studies showed that single substitutions at amino acid positions E107, E138, E176/177, or E279 differentially modulated viral virulence [22]. Our results showed that inoculation with the rJEV2 (E138) mutation increased SA14-14-2 neurovirulence to the highest level, followed by that of the rJEV1 (E107) mutation, whereas the single revertant mutation of E279 had no effect on neurovirulence. A synergistic virulence effect was observed when the E138 revertant mutation was combined with E107, but not E176/177 or E279, whereas infection with rJEV5 (E138/E107) exhibited the lowest LD_50_ (log_10_PFU) value (1.70). These results were consistent with observations that after five passages in the suckling mouse brain, the revertant mutations at E138 and E107, from JEV SA14-14-2 to those of the parental SA14 strain, increased the neurovirulence of the resulting virus [22].

Residue E107 is located within a highly-conserved hairpin motif spanning amino acids 98 to 111 in domain II [23]. This region contains a fusogenic peptide according to studies of the tick-borne encephalitis virus, the Murray Valley encephalitis virus, and the dengue type 2 virus [24,25]. Mutations in close proximity to this region alter the fusion properties of the E protein in cell culture and are associated with changes in the neurovirulence of the dengue virus and the tick-borne encephalitis virus [26,27]. Residue E138 is located in the ‘hinge’ region at the interface of domains I and II of the E protein, and mutation at this position alters E protein conformation and function. Previous studies of *Flaviviruses* indicated that mutations within this region modulate viral virulence in mice [28,29,30,31,32], thereby supporting the results of this study.

The effect of the E176/177 cluster on viral neurovirulence is interesting. In contrast to other reverse mutations that increased SA14-14-2 virulence, single substitutions in the E176/177 cluster elevated viral virulence to a lesser extent than other substitutions; however, when combined with mutations at E107 or E138, E176/177 mutations significantly decreased viral virulence. Additionally, the virulence of rJEV6 (E107/E176/E177) was lower than that observed for rJEV1 (E107) (5.69 vs. 3.97), the virulence of rJEV7 (E138/E176/E177) was lower than that observed for rJEV2 (E138) (3.64 vs. 2.89), and the virulence of rJEV9 (E107/E138/E176/E177) was lower than that of rJEV5 (E107/E138) (1.99 vs. 1.77). Therefore, we concluded that the E176/177 mutations significantly neutralized the function of E107 and E138.

Residue E279 is located in the hinge region of the E protein, suggesting a possible regulatory role in E protein function, similar to that of E138. Previous studies showed that reverse mutation of residue E279 from methionine to lysine significantly increased neurovirulence [33]. Additionally, mutations in close proximity to E279 in the Murray Valley encephalitis virus impair hemagglutination and fusion properties of the E protein and reduce neuroinvasiveness in mice [28,34]. By contrast, reverse mutation of E279 in this study did not affect SA14-14-2 neurovirulence. The neutral effect of the E279 mutation might be explained by the decreased ability of the virus harboring the mutation to infect host cells, given that inoculation with rJEV4 (E279) resulted in that formation of the smallest plaques among all tested viruses, including SA14-14-2.

A previous study reported that the molecular determinants associated with the prM-E region of the attenuated JE SA14-14-2 virus are insufficient to confer an attenuated phenotype upon the JE Nakayama virus [6]. This suggested a role for determinants located in the 5′ untranslated region and/or the capsid protein of the JE SA 14-14-2 viral genome in influencing the virulent properties of the JE Nakayama virus in mouse models. Here, we observed that the revertant rJEV11 virus, having the same eight amino acids in the E protein as the parental virulent SA14 strain, exhibited significantly lower neurovirulence and neuroinvasiveness in mice as compared with JEV SA14 (Table 2 and Table 4). These data demonstrated that mutations in viral proteins (including nonstructural protein) other than the E protein in JEV SA14-14-2 may also contribute to attenuated neurovirulence and neuroinvasiveness.

A previous report utilized a chimeric YFV/JEV SA14-14-2 virus to characterize the attenuation mechanism [35]. This chimeric virus contained the backbone of YFV and the prM and E protein sequences from the attenuated JEV SA14-14-2 strain. Consistent with our findings, Arroyo et al. [35] reported the importance of the E138 amino acid together with amino acid residues at other positions in neurovirulence attenuation; however, other results from that study differed from our findings. Arroyo et al. [35] reported that inoculation with variants harboring single reverse mutations of E107, E138, and E176/177 did not cause sickness or death in mice and that the single reverse mutation of E279 caused death in only 13% of mice. By contrast, we observed that inoculation with each of the three single reverse mutations (E107, E138, and E176/177) resulted in sickness or death in some of the mice and isolation of the revertant viruses in the brain. Additionally, the single reverse mutation of E279 did not cause sickness or death in mice. These discrepancies might be explained by the different inoculation doses used between the two studies, given that our inoculated mice became sick or died only when inoculated with >4.0 log_10_PFU of the revertants rJEV1 (E107), rJEV3 (E176/E177), and rJEV6 (E107/E176/E177), whereas the previous study used inoculation doses via i.c. of <4.0 log_10_PFU (10,000 PFU) [35]. Additionally, the results of that study did not suggest an important role for the single mutation of E107 in JEV attenuation, whereas our results provided a more detailed account of the roles of E176/177 and E279 in JEV attenuation, in combination with E138. Furthermore, Arroyo et al. [35] reported that the single substitution at E176/177 greatly enhanced virulence in combination with other mutations, whereas we observed decreased virulence associated with this mutation in combination with others. One explanation for these discrepancies might be use of a chimeric virus with YFV as the backbone in the previous study [35], whereas the JEV SA14 virus was used in this study to investigate the attenuation mechanism.

**Figure 3:**
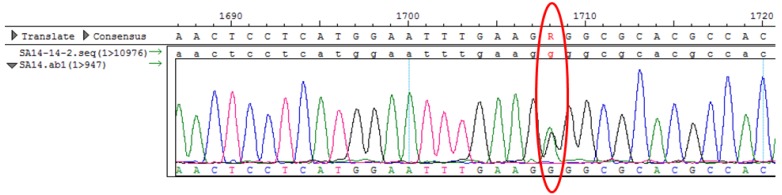
An equal proportion of two different amino acids exists at position E244 in the SA14 virus.

Yun et al. [36] showed that the passage of the JEV SA14-14-2 strain in the mouse brain was selected for mutations at the E244 position, which drastically altered the viral phenotype [36]. In this study, this amino acid position was not tested because the E244 position in the SA14 viral population harbors two different amino acids in the mouse brain (Figure 3), with glutamic acid at this position in wild-type SA14/USA and glycine at this position in the SA14/CDC and SA14/JAP strains (Table 1 and Figure 3).

The mechanisms associated with viral attenuation are complicated and involve membrane fusion [24,25], replication capacity [12], and heparin-binding activity [37]. In addition to the SA-14 E protein, our findings suggested that other regions of the viral genome likely also contribute to the attenuated phenotype. This was supported by our results showing that the virulence of the revertant rJEV11/SA14 strain remained lower than that of the parental SA14 strain, despite substitution with the intact E protein from wild-type SA14.

## 5. Conclusions

In summary, this study demonstrated that amino acids at positions E107, E138, E176/177, and E279 differentially contributed to virulence attenuation in the SA14-14-2 virus. Our findings indicated that the E138 position played the most important role in sustaining neurovirulence, but not the attenuated neuroinvasive phenotype associated with JEV SA14-14-2. Additionally, the role of the E107 position in attenuating virulence was revealed by its synergistic effect with the E138 position, although E107 alone also contributed to virulence attenuation. Compared with the E107 and E138 positions, E176/177 and E279 exhibited relatively minor roles in virulence attenuation. These results identified the key residues in the E protein involved in regulating attenuated JEV SA14-14-2 virulence, thereby elucidating the molecular mechanisms of JEV attenuation. The data presented in this study supported JEV vaccine guidelines stating that the stability of the E protein sequence should be used as the main safety indicator for the attenuated live JE vaccine (SA14-14-2 strain).

## Figures and Tables

**Table 1:** Amino acid differences in the viral E protein between Japanese encephalitis virus (JEV) strains SA14, SA14-14-2, and SA14-5-3 [3,4].

Positions in E Protein ^a^	Virulent Strain			Attenuated Strain		
	SA14/USA	SA14/CDC	SA14/JAP	SA14-14-2/PHK	SA14-14-2/PDK	SA14-5-3
E107	L	L	L	F	F	F
E138	E	E	E	K	K	K
E176	I	I	I	V	V	V
E177	T	T	T	A	T	T
E264	Q	Q	Q	H	Q	Q
E279	K	K	K	M	M	M
E315	A	V	A	V	V	V
E439	K	R	K	R	R	R

^a^ Amino acids that differ between the virulent JEV strain SA14 and the attenuated SA14-14-2 and SA14-5-3 strains are highlighted in bold letters. E177 was studied along with E176 due to their close proximity.

**Table 2:** Neurovirulence of the mutated viruses in 3-week-old mice inoculated by the i.c. route.

Viruses		LD_50_ (log_10_PFU) *
rJEV (SA14-14-2)	≥6.48	
rJEV1 (E107)	3.97	
rJEV2 (E138)	2.89	
rJEV3 (E176/E177)	≥6.43	
rJEV4 (E279) ^†^	≥6.24	
rJEV5 (E107/E138)	1.70	
rJEV6 (E107/E176/E177)	5.69	
rJEV7 (E138/E176/E177)	3.64	
rJEV8 (E138/E279)	2.82	
rJEV9 (E107/E138/E176/E177)	1.99	
rJEV10 (E107/E138/E176/E177/E279)	1.43	
rJEV11/SA14	0.66	
SA14	−0.92	

* LD_50_ (log_10_PFU) represents the plaque titers that cause death in 50% of tested mice; ^†^ Virus rJEV4 did not cause neurovirulence.

**Table 3:** AST and mortality of mice inoculated with the viruses by the i.c. route.

Viruses	No. of Dead Mice/Total No. of Mice (%)	AST (day) Mean ± SD
rJEV (SA14-14-2)	0/6 (0)	-
rJEV1 (E107)	5/6 (83.3%)	6.6 ± 0.9 ^$^
rJEV2 (E138)	6/6 (100%)	6 ± 0 ^#,$^
rJEV3 (E176/E177)	1/6 (16.7%)	11 ± 0
rJEV4 (E279)	0/6 (0)	-
rJEV5 (E107/E138)	6/6 (100%)	6 ± 0 ^#^
rJEV6 (E107/E176/E177)	3/6 (50%)	9 ± 0
rJEV7 (E138/E176/E177)	6/6 (100%)	6 ± 0 ^#^
rJEV8 (E138/E279)	6/6 (100%)	6 ± 0 ^#^
rJEV9 (E107/E138/E176/E177)	6/6 (100%)	6 ± 0 ^#^
rJEV10 (E107/E138/E176/E177/E279)	6/6 (100%)	6 ± 0 ^#^
rJEV11/SA14	6/6 (100%)	5 ± 0 *
SA14	6/6 (100%)	4 ± 0 *

^#^ *p* = 1, compared with each other; * *p* ≤ 0.05, compared with each other; ^$^ *p* ≤ 0.05, compared with each other.

**Table 4:** Neuroinvasiveness of the revertant viruses in 3-week-old mice inoculated by the s.c. route.

Inocula	LD_50_ (log_10_PFU)
rJEV (SA14-14-2)	≥6.14
rJEV1 (E107)	≥7.32
rJEV2 (E138)	≥6.20
rJEV3 (E176/E177)	≥6.93
rJEV4 (E279)	≥6.74
rJEV5 (E107/E138)	≥6.54
rJEV6 (E107/E176/E177)	≥6.71
rJEV7 (E138/E176/E177)	5.76
rJEV8 (E138/E279)	6.01
rJEV9 (E107/E138/E176/E177)	5.40
rJEV10 (E107/E138/E176/E177/E279)	5.53
rJEV11/SA14	3.17
SA14	1.86
